# PCOS in Adolescents—Ongoing Riddles in Diagnosis and Treatment

**DOI:** 10.3390/jcm12031221

**Published:** 2023-02-03

**Authors:** Blazej Meczekalski, Olga Niwczyk, Anna Kostrzak, Marzena Maciejewska-Jeske, Gregory Bala, Anna Szeliga

**Affiliations:** 1 Department of Gynecological Endocrinology, Poznan University of Medical Sciences, 60-535 Poznan, Poland; 2 UCD School of Medicine, University College Dublin, D04 V1W8 Dublin, Ireland

**Keywords:** polycystic ovary syndrome, PCOS, adolescent

## Abstract

Polycystic ovary syndrome (PCOS) is one of the most common endocrine disorders in women of reproductive age. A diagnosis of PCOS is established when a patient exhibits two of three Rotterdam criteria: oligoovulation or anovulation, excess androgen activity, and polycystic ovarian morphology. The pathogenesis of PCOS, as it affects adolescents, is often discussed in terms of a “two-hit” theory. This refers to a stepwise process in which the first “hit” is an inborn congenitally programmed predisposition, while the second “hit” arises from a provocative factor such as insulin resistance. The dynamic physiological and anatomical changes which occur in puberty make for a challenging diagnosis in this group of patients. It is important to be mindful of the physiological particularities in adolescence which often mimic the symptoms of PCOS. In their first-year post-menarche, approximately 75% of menstruating adolescents report their cycle to last between 21–45 days. Recent studies have shown that regular menstrual cyclicity is only achieved within 2–3 years post-menarche. Anovulation, as a crucial diagnostic element for PCOS, features in about half of early-post-menarchal adolescents. Hirsutism and acne are the most common clinical manifestations of hyperandrogenism, and mild features are developed by most adolescents as a result of elevated androgen levels. Distinguishing between a pathological sign and normal features of maturation is often difficult. A polycystic ovarian morphology (PCOM) through ultrasound has been found in up to 40%, 35%, and 33.3% of patients when assessed at 2, 3, and 4 years, respectively, after menarche. PCOM in adolescence is not associated with future abnormalities in ovulatory rate or menstrual cycle duration. For this reason, international guidelines recommend against the use of pelvic ultrasound until 8 years post-menarche. The primary aim of management is focused mainly on improving hormonal and metabolic status, the prevention of future comorbid complications, and generally improving the overall quality of life in young women with PCOS. Considerable controversy surrounds the choice of optimal pharmacological treatment to address PCOS in adolescents. Reliable studies, which include this sub-section of the population, are very limited. There is a lack of robust and reliable trials in the literature addressing the use of combined oral contraceptives. Further work needs to be undertaken in order to provide safe and effective care to the adolescent population in this regard.

## 1. Introduction

Polycystic ovary syndrome (PCOS) is the most common endocrine disorder in women of reproductive age. Criteria of diagnosis consist of oligoovulation and/or anovulation, excess androgen activity, and polycystic ovary morphologies. While the exact etiology is yet to be identified, major contributing factors are suspected to include environmental factors, the LH–theca interstitial hypothesis, and assorted genetic variants. Considering its prevalence and likely intrinsic nature, establishing specific diagnostic criteria for PCOS in adolescence has remained a challenge. The dynamic physiological and anatomical changes which occur during puberty make a diagnosis in this group difficult. Hyperandrogenism, insulin resistance, anovulation, irregular menstrual cycles, and polycystic ovarian morphology are all traits of female reproductive axis maturation. 

Additionally, managing PCOS in adolescent patients is challenging. The task is not made any less difficult by its obscure pathophysiology, the ongoing controversy surrounding diagnosing PCOS in adolescents or even the comparatively limited number of studies which specifically focus on PCOS in adolescents. The aim of managing such patients focuses mainly on the improvement of hormonal and metabolic status, the prevention of future comorbid complications, and generally improving the quality of life in young women with PCOS. This review aims to summarize the latest data on the diagnosis and management of adolescent patients with PCOS. 

## 2. Methods

This narrative review was conducted using a search in several major databases, including PubMed, ScienceDirect, Excerpta Medica Database, UpToDate, and Cochrane Library. The search was conducted between 1 September 2022 and 15 October 2022. The authors investigated the available data from clinical studies, review articles, and meta-analyses published in English until June 2022 which reported on specifics in PCOS pathogenesis, diagnosis, and management in adolescents. The following MeSH terms were used, alone or in combination: ‘PCOS’, ‘adolescent’, ‘adolescence’, ‘young’, and ‘polycystic ovary syndrome’. All publications were critically evaluated by the authors, including those more specifically related to the theme. Moreover, reference lists of included articles were manually screened to identify additional studies. 

## 3. Risk Factors for Developing PCOS

Although Stein and Leventhal first recognized and published their observations on PCOS in 1935, the causes and risk factors of this condition remain largely unclear to this day [1]. Various iterations of diagnostic criteria and guidelines have been proposed to date, and numerous publications report on risk factors and the development of PCOS [2]. While the exact etiology has yet to be identified, major contributing factors are suspected to include environmental factors, the LH–theca interstitial hypothesis, and assorted genetic variants. These have all featured as elements in the development of PCOS, although denoting the extent and importance of each element still requires further investigation.

The impact that a patient’s environment has on their development has yet to be properly studied. It is understood, however, that toxins and social economic status may feature as strong determinants in the prevalence of PCOS. Some studies even report factors as broad as geography—climate, altitude, and terrain are contributory. Other studies suggest that variants in ethnicity and race are involved. The scope of most studies, however, does not consider these macroelements. Most investigations revolve around lifestyle, physical activity, and dietary habits (insulin resistance and endocrine disruptors) [3]. A lot of recent investigations have revealed that oxidative circulating markers are significantly increased in patients with PCOS compared with the norm and are considered a potential inducement of PCOS pathogenesis [4,5]. Additionally, the significant evidence for the correlation between the intestinal microbiome and the development of metabolic disorders has led to the postulation of the hypothesis that alterations in the microbiome are also involved in the genesis of PCOS [6].

A familial element in the prevalence of PCOS is well established in that development and symptoms have a hereditary element. For this reason, a mutation or polymorphism hypothesis has been proposed and developed over the years.

Genetics, as a basis for PCOS, proposes that the variance in the gene coding for elements of steroidogenesis, proteins related to steroid hormone function as well as gonadotrophin release and regulation underpin the disorder. It would also imply that adipose tissue metabolism and insulin secretion play a role in the process [7]. The most important genes to consider in the etiology of PCOS include CYP11A1, CYP17A1, and CYP19 A1. Studies have suggested that epigenetic changes during fetal life impact the possibility of developing PCOS in the future.

Patients with PCOS have elevated serum LH levels which stimulate steroidogenesis in theca cells producing androgens. Steroidogenesis is the process by which cholesterol is converted to bioactive compounds by cells of steroidogenic-specific organs. The process is conducted by steroidogenic enzymes including steroid reductases such as hydroxysteroid dehydrogenase (HSDs) and enzymes of the cytochrome P450 (CYPs) family. In the ovaries, cholesterol is converted to pregnenolone by CYP11A1 under the influence of the luteinizing hormone (LH). Pregnenolone in turn is hydroxylated first to 17-hydroxypregnenolone before conversion to dehydroepiandrosterone (DHEA) by CYP 17A1 [8]. In granulosa cells of the ovary, CYP19A1 aromatase (also known as estrogen synthetase) catalyzes the production of estrogen. The CYP19A1 gene codes for aromatase, which is expressed in many tissues and catalyzes multiple reactions including those involved in the synthesis of steroids, androgens, glucocorticoids, and progestins.

Hyperandrogenism is one of the most prominent clinical manifestations of PCOS [9]. Although it is unclear whether hyperandrogenism is a cause or symptom of PCOS, an enzyme deficiency at any stage in the steroidogenic pathway is prognostic [10]. Neuropeptide and neurohormone functions are also heavily involved from the hypothalamus, with disruption at any point along this process potentially leading to the development of PCOS [2].

The pathogenesis of PCOS in adolescents is often discussed in terms of a “two-hit” theory. This refers to a stepwise process in which the “first hit” arises from a congenitally programmed predisposition that is either hereditary (genetic) or acquired (e.g., maternal androgens). The “second hit “arises from a provocative factor, usually insulin resistance. PCOS develops when both hits are triggered [2]. 

As PCOS itself is such a wide and multifactorial condition, future studies are required to better identify its associated risk factors. As larger and more robust studies are undertaken, we will hopefully have a better understanding of the many genetic and environmental factors involved in this condition, which will lead to more accurate prediction, prevention, and proper treatment for women with PCOS [2].

## 4. Criteria of Diagnosis—Considerations for Young Patients

For authors considering its prevalence and likely intrinsic nature, establishing specific diagnostic criteria for PCOS in adolescence has remained a challenge. The dynamic physiological and anatomical changes which occur during puberty make a diagnosis in this group difficult. Hyperandrogenism, insulin resistance, anovulation, irregular menstrual cycles, and polycystic ovarian morphology are all traits of female reproductive axis maturation (Table 1). 

National Institute of Health (NIH) criteria were established in 1990 as a result of experts’ efforts to develop standard diagnostic criteria for PCOS. NIH criteria included clinical or biochemical evidence of hyperandrogenism and ovulatory dysfunction [11]. PCOM was not incorporated into these criteria because it was already known at that time that polycystic morphology of the ovaries is observed in 20–30% of healthy women [12]. NIH criteria served as a standard for PCOS diagnosis for more than a decade until 2012 when NIH recommended using the Rotterdam criteria.

Rotterdam criteria were another step forward in PCOS diagnosis, with PCOM added as a diagnostic criterion. According to the Rotterdam consensus, PCOS is defined by the presence of two of three of the following criteria: oligo/anovulation, hyperandrogenism, and polycystic ovaries [13].

Despite the fact that both NIH and Rotterdam criteria were created based on observation in adult women, in clinical practice, these same criteria have been extrapolated for use in adolescents. Over the last decade, international adolescent consensus promoted the use of NIH diagnostic criteria over the Rotterdam criteria [14]. The Rotterdam criteria have been considered inappropriate for adolescent girls as many healthy juveniles have polycystic ovarian morphology in the early years post-menarche [15].

Being aware of the fact that features of normal pubertal development overlap with adult diagnostic criteria, separated guidelines in adolescents are still required.

**Table 1:** Differences in criteria for PCOS diagnosis in adolescent patients.

Criteria Definition	Ibáñez et al., 2017 [16]	Peña et al., 2020 [17]
Menstrual Irregularity	Irregular menses/oligomenorrhea 2 years post-menarche. Menstrual cycles > 90 days 1-year post-menarche. Primary amenorrhea in girls with completed puberty.	Irregular menstrual cycles < 1-year post-menarche represents a normal pubertal transition. >90 days for any one cycle > 1-year post-menarche. Cycles< 21 or >45 days >1 to <3 years post-menarche. Cycles < 21 or >35 days 3 years post-menarche. Primary amenorrhea by age 15 or >3 years post-thelarche.
Hyperandrogenism	Evidence of hyperandrogenism: Biochemical—no clear testosterone concentration cut-offs; confirmation of biochemical hyperandrogenism in symptomatic adolescents. Clinical—hirsutism and/or moderate or severe inflammatory acne, especially if unresponsive to topical therapy.	Evidence of hyperandrogenism: biochemical—no clear testosterone concentration cut-offs; calculated free testosterone, free androgen index, or bioavailable testosterone evaluation with high-quality assays. clinical—hirsutism assessed with standardized visual scales e.g., the Ferriman–Gallwey scale and/or moderate or severe comedonal acne (i.e., 10 or more facial lesions), or moderate to severe inflammatory acne.
Polycystic Ovary on Ultrasound	The presence of PCOM in an adolescent who does not have hyperandrogenism/oligo-anovulation does not indicate a diagnosis of PCOS.	Pelvic ultrasound should not be used for the diagnosis of PCOS in those with a gynecological age of <8 years.

### 4.1. Androgens

When assessing for hyperandrogenism, both clinical and biochemical pictures need to be considered. Numerous studies have reported on strategies for diagnosing PCOS-related hyperandrogenism, however, only one such study has specifically examined adolescent patients. This study by Villarroel et al. proposes that calculating free testosterone, free androgen index, and bioavailable testosterone are the most relevant markers of hyperandrogenism [18]. The authors compared a group of hirsute girls presenting with oligomenorrhea and a group of non-hirsute girls with regular cycles. They concluded that a free androgen index ≥ 6.1 and testosterone ≥ 2.4 nmol/L were the most diagnostically reliable parameters for PCOS. Neither the guidelines published by Ibáñez L. et al. for the ICPE nor the international guideline published by Peña A. S. et al. presented clear cut-off points for serum androgen levels when used during diagnosis. Both recommendations do highlight the importance of diagnostic accuracy and the accuracy of different types of assays. The use of consistent analysis methods with high sensitivity such as liquid chromatography–mass spectrometry and extraction/chromatography immunoassays should be used, especially in cases where clinically significant hyperandrogenism is not detected. 

West et al., [19] investigated whether adolescent hyperandrogenemia is an early sign of PCOS. Their prospective cohort study was foundational in establishing the link between elevated androgen levels at 16 years and symptomatic PCOS and fertility issues at 26 years. This information reinforces that adolescents with isolated hyperandrogenism should not be diagnosed with PCOS immediately but instead considered at risk of developing PCOS in the future. Other androgens such as dehydroepiandrosterone sulfate (DHEAS) or androstenedione were not included in the diagnostic criteria but can be useful in excluding alternative causes of hyperandrogenism. 

The most common clinical manifestation of hyperandrogenism is hirsutism and acne. During puberty, many adolescents develop mild features which are characteristic of elevated androgen levels. Distinguishing between a pathological sign and normal features of maturation can be difficult. A comprehensive history and physical examination are essential to establish an accurate differential diagnosis. Where mild comedonal acne is common in adolescent girls, severe acne is considered an indicator of underlying PCOS. Currently, there is no established consensus in evaluating the severity of acne; however, it is generally accepted that a larger number of comedonal lesions (usually 10 or more simultaneously) which are resistant to topical medications and cause residual scarring is considered to be severe acne.

Assessing hirsutism in a standardized manner is easier in part due to semi-objective visual scoring systems. The modified Ferriman–Gallwey score (mFG) evaluates nine body areas (upper lip, chin, neck, chest, upper and lower abdomen, thighs, upper and lower back) with a score of 0–4 assigned to the extent of terminal hair growth. The obvious limitation of this tool has been the popularity and widespread accessibility of cosmetic hair removal techniques, which often effectively masque symptomology and impair assessment. The mFG score is primarily criticized, however, for its lack of defined cut-off points or means of calibration as hirsutism largely depends on ethnic background and varies significantly across different regions. The currently accepted cut-off point for hirsutism was established using a random selection of women in which an mFG score greater than 6–8 constituted the 95th percentile of hair growth for the group. It is important to note that these cut-offs are made based on studies that include only adult women and that no studies have been undertaken to define the optimal cut-off for adolescents. Hirsutism in adolescence is less prominent compared to adulthood because hair growth only becomes thick and coarse with increasing duration of androgen exposure. Hirsutism developing in the presence of increased serum androgens is a lengthy process [20]. The high prevalence of hirsutism among adolescents with PCOS has been confirmed in multiple studies [21,22].

Male pattern hair loss or alopecia is another feature of clinical hyperandrogenism which manifests as a reduction in hair density over the central area of the scalp while the frontal hairline remains generally well conserved. A study by Carmina et al. [23] reported observing alopecia in 20–30% of adult patients with PCOS. Regrettably, there are no equivalent adolescent studies evaluating alopecia in the context of PCOS. Overall, hair loss alone is not recommended as a diagnostic tool for PCOS in this group. 

Clinical hyperandrogenism, whether in the context of PCOS or otherwise, needs to be carefully examined and interpreted within the context of each patient since cutaneous findings can have a potentially severe negative psychosocial impact and negatively affect a patient’s quality of life.

### 4.2. Menstrual Cycle

It is nearly impossible to distinguish menstrual cycle irregularity as a result of PCOS from the cycle irregularity typically seen in the first years post-menarche. Regular cyclicity of the menstrual cycle is often achieved only in the years that follow menarche; an exact timeframe within which a regular pattern of menstrual bleeding is established is as yet undefined. Approximately 75% of menstruating adolescents report their cycle to be between 21–45 days in the first-year post-menarche. Recent studies have shown that most adolescents attain regular menstrual cyclicity within 2–3 years post-menarche. Numerous studies over the years have examined menstruation patterns in postmenarchal girls. A 2020 Danish study by Assens M. et al. observed that at 3 years following menarche, the majority of subjects studied had developed a regular menstrual cycle while only 6.3% exhibited persistent oligomenorrhea [24]. Similarly, an Italian population-based study investigating menstrual cycle characteristics in a group of 3783 adolescents found that within the fourth gynecological year, the prevalence of irregular menstrual cycles stabilizes at under 10% [25]. Based on these data, the diagnostic difficulties in determining PCOS in adolescence are generally understood. Two separate guidelines proposed independently by Witchel S. et al. [26] and Ibáñez L. et al. [16] recommended waiting at least two years after menarche before diagnosing oligomenorrhea when cycles persist longer than 45 days. In their guidelines, Peña et al. [17] provide specific definitions for menstrual irregularities at time intervals post-menarche and take into consideration normal physiological milestones during adolescence. This proposed guideline assumes that irregular cycles are normal in the first-year post-menarche. In years 1–3, a menstrual cycle of <21 and >45 days is defined as irregular, while in postmenarchal years 3 and later, cycles of <21 or >35 days or <8 cycles per year are considered irregular. All three research groups agree that menstrual cycles >90 days at 1-year post-menarche are considered a menstrual cycle disturbance. 

Despite the difficulties in diagnosing PCOS during adolescence, it remains important to identify those adolescents who are at risk. Studies have indicated that irregular menstrual cycles during puberty are predictive of future PCOS development. In 2021, Caanen et al. [27] reported that the prevalence of developing PCOS in adulthood increased to 22.5% in girls with oligomenorrhea during adolescence compared to 5.1% in subjects who reported regular cycles. These data are supported by a previous Finnish study in which West et al. [19] observed that the incidence of persistent menstrual irregularity and PCOS in adulthood was significantly higher in women who reported menstrual irregularity in adolescence when compared to women who reported regular menstrual cycles. It is, therefore, advisable that appropriate follow-up is provided to any adolescent who presents with isolated irregular menstruation.

Ovulation is a pinnacle point of the menstrual cycle. Anovulation, on the other hand, is a central characteristic of PCOS, which also differs in adolescent girls compared to adult women. The Rotterdam consensus, which led to the diagnostic criteria used for PCOS in adults, attributes great importance to anovulation as the identifying symptom. While this is a crucial diagnostic element, it is also important to keep in mind that about half of early adolescents will have anovulatory cycles [28]. In this context, using serum progesterone levels in a single menstrual cycle, as is often the method used in adult patients, is not recommended to determine ovulation in adolescents. 

### 4.3. PCOM

Despite including polycystic ovary morphology (PCOM) in pelvic ultrasound as a diagnostic criterion in the Rotterdam criteria for the diagnosis of PCOS in adults, there remains strong consensus against using pelvic ultrasound in the diagnosis of PCOS in adolescents [17]. This reluctance stems from the fact that PCOM can be a transient condition occurring in healthy young women, while the common use of ultrasound can easily lead to PCOS over-diagnosis in adolescents. Codner et al. [29] studied the association of PCOM with ovarian function in adolescents. They observed PCOM in ultrasound in 40%, 35%, and 33.3% of patients when performed at 2, 3, and 4 years after menarche, respectively. Most importantly, they observed that PCOM was not associated with abnormalities in ovulatory rate or menstrual cycle duration. For this reason, the international guidelines recommend avoiding the use of pelvic ultrasound up until 8 years post-menarche. A gynecological age of 8 years was determined as the cut-off based on a normative model of the ovarian volume published by Kelsey et al. [30] who determined that average peak ovarian volume is reached by age 20.

The second argument proposed against including PCOM in the diagnostic criteria for PCOS in adolescents is that the majority of ultrasound examinations in this patient group are conducted transabdominally and not transvaginally. This difference in modality affects the accuracy of findings and measurements. Very few studies have evaluated the diagnostic value of transrectal ultrasonography in adolescent patients with PCOS. In one such study, however, Sun et al. [31] concluded that the transrectal modality is more reliable than transabdominal in adolescents. Ovarian stromal area and stromal area to total area ratio (S/A ratio) were calculated and found to be significantly greater in patients with PCOS compared to controls. The authors went on to propose the S/A ratio as an ultrasonographic diagnostic marker for PCOS. Ultimately, this one study does not provide sufficient evidence on which to base new diagnostic guidelines, and further studies are required to establish the limitations of transrectal US.

Although pelvic ultrasound is not indicated for the diagnosis of PCOS in adolescents, it is still a useful tool to evaluate other possible uterine or ovarian abnormalities in the differential.

Summary: 

The dynamic physiological and anatomical changes which occur during puberty make a diagnosis in this group particularly difficult.

- Approximately 75% of menstruating adolescents report irregular menstrual cycles in the first years post-menarche.
- Most adolescents attain regular menstrual cyclicity within 2–3 years post-menarche.
- Distinguishing between pathological acne and normal features of maturation can be difficult.
- It is recommended to avoid the use of pelvic ultrasound up until 8 years post-menarche.

## 5. Metabolic Problems

PCOS is host to numerous metabolic comorbidities including insulin resistance (IR), hyperinsulinemia, impaired glucose tolerance, type 2 diabetes mellitus (T2DM), gestational diabetes, hypertension, non-alcoholic fatty liver disease (NAFLD), dyslipidemias, metabolic syndrome, and increased cardiovascular risk [32]. 

Lean girls with PCOS were found to have decreased peripheral insulin sensitivity, abnormal glucose disposal, relative postprandial hyperinsulinemia, and increased hepatic fat compared to normal-weight controls. At the tissue level, women with PCOS express insulin resistance in the liver, adipose, and muscle tissues [33]. A study conducted by Arslanian S. et al. assessed for impaired glucose metabolism in obese adolescents with PCOS. They observed that glucose intolerance was associated with decreased first-phase insulin secretion, a decreased glucose disposition index, and increased hepatic glucose production. This study underlines the importance and necessity of early diagnosis as these metabolic abnormalities are precursors to type 2 diabetes and are present early in the course of PCOS [34].

Abnormal glucose metabolism features in 18.2% of young patients with PCOS. IGT is the most commonly observed abnormality occurring with equal frequency in obese and non-obese adolescents. When compared, non-obese adolescents with IGT had similar mean 2-h insulin, high-density lipoprotein, C-reactive protein, and testosterone levels to obese adolescents, despite marked differences in BMI (*p* < 0.001) and % body fat (*p* = 0.002) [35].

The risk of metabolic complications such as gestational diabetes, IGT, and DM-II increases 5-fold in Asia, 4-fold in the Americas, and 3-fold in Europe in the presence of PCOS [36]. Patients burdened with these comorbidities experience a decrease in overall quality of life and lifespan. It is therefore imperative that appropriate management of PCOS be implemented to prevent future life-threatening complications [33]. 

Glycemic status should be evaluated at baseline in all women with a new diagnosis of PCOS. As mentioned above, it is particularly important for healthcare professionals to bear in mind that the prevalence of impaired glucose tolerance, gestational diabetes, and type-2 diabetes are significantly increased in PCOS [36]. At the very least, an oral glucose tolerance test (OGTT), fasting plasma glucose, or HbA1c should be performed every one to three years and adjusted anent other diabetic risk factors. In high-risk women with PCOS, such as those presenting with a BMI > 25 kg/m_2_ (or >23 kg/m^2^ in Asian ethnicities), a history of impaired fasting glucose, impaired glucose tolerance or gestational diabetes, family history of type-2 diabetes mellitus, hypertension, or those identified as a high-risk ethnicity, an OGTT is recommended [36].

## 6. Mood disorders

**Figure 1:**
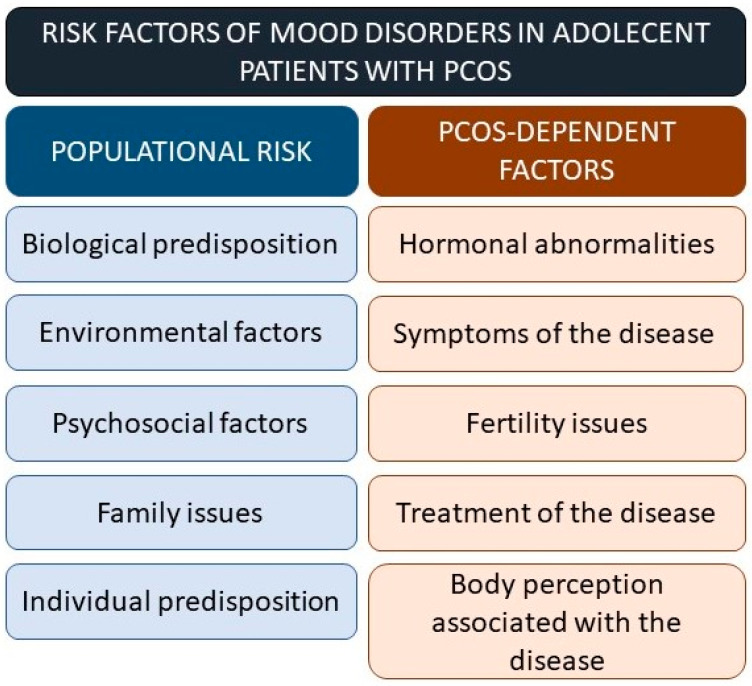
Risk factors for mood disorders in adolescent patients with PCOS.

Mood disorders are very common in patients with PCOS [37]. Many factors, both related and unrelated to the disease may contribute to the development of the disease (Figure 1). Depression and anxiety are the most common psychological comorbidities observed in young patients with PCOS. Eating disorders, psycho-sexual dysfunction, negative body image, and reduced QoL are also frequently present. Young patients with PCOS are at a higher baseline risk for depression as well as a higher risk for the de novo development of depressive symptoms [32].

Adolescent girls with PCOS are 2.4 times more likely to suffer from depression than healthy girls of the same age [38]. In a study conducted by Coban O. et al., quality of life (QL) was measured using the Pediatric Quality of Life Inventory (PedsQL) and self-esteem was measured using the Rosenberg self-esteem scale (RSES). The rate of pre-existing psychiatric diagnosis was higher in the PCOS group compared to the control group (*p* < 0.05). Twenty-one percent of patients in the PCOS group met the criteria for a diagnosis of a depressive disorder. Major depressive disorder was the most common single diagnosis. Despite these findings, there were no significant differences observed between the PCOS and control groups in terms of RSES and PedsQL scores. The authors also noted that no significant relationship was observed between QL or body image and hirsutism, acne, or body mass index [39]. 

Similar findings were observed by Sari S et al., where the rate of psychiatric disorders was significantly higher in the PCOS group compared to a group of control participants (32% vs. 13.5%, *p* = 0.046). Similarly, major depressive disorder was the most common diagnosis, while no significant relationship was observed between obesity, hirsutism, or insulin resistance and psychiatric disorders in the PCOS group [40].

In a meta-analysis examining mood in PCOS patients, authors Cooney L et al. observed that women with PCOS were more likely to exhibit any depressive symptoms (OR: 3.78; 95% CI: 3.03–4.72; 18 studies) and moderate/severe depressive symptoms (OR: 4.18; 95% CI: 2.68–6.52; 11 studies). Women with PCOS were also more likely to exhibit any anxiety symptoms (OR: 5.62; 95% CI: 3.22–9.80, 9 studies) and moderate/severe anxiety symptoms (OR: 6.55; 95% CI: 2.87, 14.93; 5 studies). When subjects were matched for BMI, women with PCOS still had higher odds of expressing both depressives (OR: 3.25; 95% CI 1.73–6.09; 4 studies) and anxiety symptoms (OR: 6.30, 95% CI: 1.88–21.09; 3 studies) [41].

It is suspected that obesity, hirsutism, and insulin resistance may exacerbate depressive symptoms and other psychiatric disorders in the PCOS population. Women who were diagnosed with concurrent PCOS and depression were also found to have a higher mean value for age, BMI, hirsutism score, and IR, while women with concurrent PCOS and anxiety had higher mean values of BMI, hirsutism score, and free testosterone (*p* < 0.05 for all comparisons). In their study, Coban et al. found no significant relationship between QL and body image related to hirsutism, acne, and body mass index, while Sari et al. observed no significant relationship between obesity, hirsutism, or insulin resistance with any psychiatric disorders in PCOS patients. 

Ding, R. et al. are attempting to calculate the absolute risk of depression as an outcome in adolescents with PCOS. This protocol is aimed at constructing and validating a warning model for depression in adolescents with PCOS. The protocol includes healthy adolescent girls as a control group and adolescent patients with PCOS as the experimental group. The assessment model includes several general factors such as individual susceptibility, biological factors, and psychosocial environmental factors of depression in adolescence as well as specific pathological, illness perception, diagnosis, and treatment factors. Symptom-related factors of PCOS as well as depression as an outcome are also included. The model should establish a basis for the prevention of depression and support control strategies which will have important theoretical and practical implications [38].

It is also important to consider that infertility in patients with PCOS can be exacerbated by emotional and psychiatric stressors due to illness which propagates a subsequent negative spiral of fear of future infertility [42].

Gender diversity is a facet of adolescent health, the psychological impact of which we have only recently started to appreciate. Schweisberger C. et al. [43] examined the prevalence of gender diversity in youth among adolescents with PCOS. Gender diversity in this context was defined as patients who self-reported their gender identity as male, fluid/both, or nonbinary. Self-declared gender diversity was observed more commonly in patients who met diagnostic criteria for PCOS at 7.6% among the study cohort compared to 1.8% in non-PCOS youth (*p* = 0.01). Gender diversity was associated with a higher hirsutism score (*p* < 0.01), but not with higher androgen levels. Depression and anxiety were higher in the gender diversity PCOS subgroup when compared to cisgender youth in the PCOS group (100% vs. 37.6%, *p* < 0.01 and 77.8% vs. 35.8%, *p* = 0.03, respectively). These findings suggest that routine gender identity screening in comprehensive adolescent PCOS programs could benefit patients in the tailoring of appropriate treatment to those who may wish to support a transmasculine identity [43].

## 7. Management of PCOS

Managing PCOS in adolescent patients is challenging. The task is not made any less difficult by its obscure pathophysiology, the ongoing controversy around diagnosing PCOS in adolescents, or even the comparatively limited number of studies which specifically focus on PCOS in adolescents [44].

The aim of managing such patients focuses mainly on the improvement of hormonal and metabolic status, the prevention of future comorbid complications, and generally improving the quality of life in young women with PCOS. 

Management in all cases of PCOS starts with lifestyle changes (weight loss and increased physical activity). It is estimated that 40–70% of adolescents with PCOS are overweight or obese [32]. It is, therefore, necessary in all patients to encourage positive lifestyle modification from the start and to continue these healthy lifestyle habits as an adjuvant should further therapy be needed. Moderate or vigorous physical activity is recommended for at least 30–60 min each day. Weight loss can decrease BMI and FG, while physical activity has an overall positive effect on menstrual cycle regulation [45].

Once lifestyle changes are undertaken, pharmacological treatment options can be considered. Estroprogestins, combined oral contraceptives (COCP), antiandrogens, and metformin are all useful tools in PCOS [17]. The use of COCP is generally regarded as first-line pharmacotherapy in adolescents. The goal of COCP use is to improve menstrual regularity and to modulate hyperandrogenemia. The use of any OCP brings with it the inherent risk of side effects. As such, starting such medication should be carefully considered and the decision process shared with the patient [16]. 

COCPs containing 20–35 mcg of ethinylestradiol and progestin are recommended. Progestins with antiandrogenic activity such as drospirenone or norgestimate should be considered. Combination therapy via a transdermal patch or vaginal ring can also be considered. Despite their low incidence of general side effects, the use of progestin-only contraception is not ideal in this group of patients. Due to their lack of estrogen component, they do not exhibit the same increase in SHBG and subsequent decrease in free androgen index [16].

If there is little or no improvement with the use of a COCP, antiandrogen strategies can be initiated. Commonly used antiandrogens include spironolactone, cyproterone acetate, and finasteride. Spironolactone acts as a partial androgen receptor agonist which has been shown to effectively decrease mFG scores. Antiandrogen use should be initiated in combination with an OCP for contraception due to the risk of teratogenic feminization in male fetuses. There is little in the way of publications, however, to evaluate the use of this strategy in adolescents [46]. Cyproterone acetate is an effective and strong progestin with antiandrogenic activity. The use of this progestin, however, has a risk of associated hepatotoxicity and meningioma. Finasteride is a 5-alpha reductase inhibitor that has been claimed to decrease serum dihydrotestosterone levels by up to 50–60%. Similar to spironolactone, the number of studies in which finasteride is used in adolescents is limited. Provisional data are available, however, which suggests that finasteride may have a negative impact on bone mineral density [47].

In adolescent cases of PCOS where lifestyle modification is inadequate in curbing obesity and insulin resistance, or if personal attitudes preclude the use of OCPs, metformin can be considered. The use of metformin in adolescents remains controversial, however, and its use is generally limited to cases having a BMI higher than 25 in conjunction with a COCP. Metformin has been shown to promote ovulation and decrease serum testosterone levels in non-obese adolescents with PCOS and hyperinsulinemia [16,48]. Side effects of metformin include gastrointestinal symptoms and lactic acidosis, while a major disadvantage of its use is the relapse of symptoms upon discontinuation of treatment. The use of additional insulin-sensitizing drugs such as thiazolidinediones has also been proposed. The use of these in adolescent women with PCOS is also not without controversy.

The hunt for new therapeutic options which can be used in the treatment of PCOS in adolescents is ongoing. Novel treatment approaches can be promising, but assuring safety in this patient population is paramount [17]. Trent et al. [49] have reported improvements in metabolic (insulin resistance) and hormonal (testosterone levels) parameters with the use of N-acetylcysteine in young women with PCOS. As an added benefit, it is very well tolerated and has a remarkably light side-effect profile, a very important characteristic should it be used for prolonged treatment. Preparations containing inositols (myoinositol and chiro-inositol) have shown promising results in promoting weight loss and menstrual cycle regulation in adolescents with PCOS [50]. It is speculated that vitamin D supplementation may exert a beneficial effect on insulin sensitivity and menstrual regularity [51]. Amr et al. [52] have reported that chromium supplementation may be beneficial in addressing the symptoms of PCOS. They report improvements in insulin resistance and menstrual irregularity. Additionally, it has been shown to decrease serum-free testosterone. Studies have also suggested the possible positive effect of carnitines (L-carnitine and N-acetyl-L-carnitine) in PCOS. Carnitines facilitate the transport of long-chain fatty acids into mitochondria where they undergo fatty acid oxidization to produce energy. They also remove products of metabolism from the cell cytoplasm. Genazzani AD et al. [53] evaluated a combination of integrative compounds (acetyl-L-carnitine, L-carnitine, L-arginine, and N-acetylcysteine) in women with PCOS and hyperinsulinemia. Following 24 weeks of treatment, the authors reported a significant improvement in metabolic profiles. 

Cosmetic interventions to address excessive hair growth can include bleaching, chemical epilation, plucking, waxing, shaving, electrolysis, and laser hair removal. Although cosmetic interventions can help promote quality of life, they do not address the underlying pathology. Very few reliable trials have been undertaken to assess these interventions in adolescents with PCOS. 

There is considerable controversy in choosing an optimal pharmacological treatment to address PCOS in adolescents. The number of reliable studies available which include this sub-section of the population is limited. There is a particular lack of robust and reliable trials in the literature addressing the use of combined oral contraceptives. Further work needs to be undertaken to provide safe and effective care to the adolescent population in this regard. 

## Data Availability

Data sharing not applicable.
